# Supplementary material for: The Evolution and Characterization of the RNA Interference Pathways in Lophotrochozoa
Source: Genome Biol Evol. 2024 May 7;16(5):evae098. doi: 10.1093/gbe/evae098 (PMC11114477; doi:10.1093/gbe/evae098)
Supplement: evae098_Supplementary_Data [file evae098_supplementary_data.zip › Supplementary_materials.zip!/selected_miRNAs/NoGe_selected_miRNAs/mirdeep_pdf_files/NMRB01000824.1_Notospermus_geniculatus_strain_Ushimado_scaffold824,_whole_genome_shotgun_sequence_21795.pdf]

Provisional ID	:	NMRB01000824.1_Notospermus_geniculatus_strain_Ushimado_scaffold824,_whole_genome_shotgun_sequence_21795
Score total	:	121087.7
Score for star read(s)	:	3.9
Score for read counts	:	121083.1
Score for mfe	:	1.3
Score for randfold	:	0
Score for cons. seed	:	-0.6
Total read count	:	237511
Mature read count	:	237446
Loop read count	:	0
Star read count	:	65

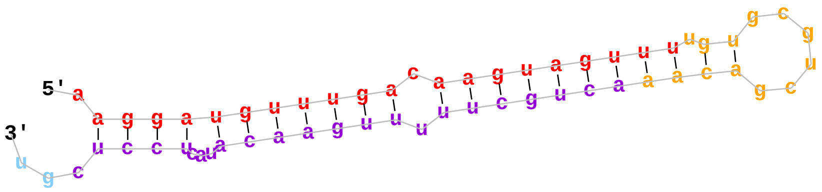

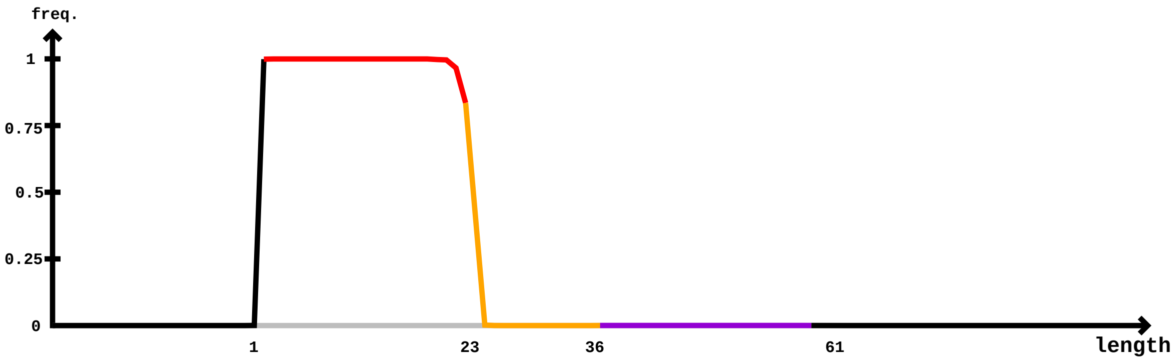

Mature **Star**

5'- aaccuugguauaugaaacguaaggauguuugacaaguaguuuugugcgucgacaaacugcuuuuugaacauacuccucguuucuuuaccgagucugcuuauugcuucacgug	-3'	obs		
aaccuugguauaugaaacguaaggauguuugacaaguaguuuugugcgucgacaaacugcuuuuugaacauacuccucguuucuuuaccgagucugcuuauugcuucacgug		exp		
...(((((((...((((((..(((((((((((.(((((((((.((......)))))))))))).)))))))...))))))))))..)))))))...((.....))........	reads		mm	sample
..................guaaggauguuugacaaguaguu...	1		0	Fm3
...................Caaggauguuugacaagua..	4		1	Fm3
...................uaaggauguuugacaagua..	1		0	Fm3
...................uaaggauguuugacaaguagu..	3		0	Fm3
...................uaaggauguuugacaaguaguu...	7		0	Fm3
...................Caaggauguuugacaaguaguu...	6		1	Fm3
...................Caaggauguuugacaaguaguuu..	15		1	Fm3
...................Aaaggauguuugacaaguaguuu..	1		1	Fm3
....................Uaggauguuugacaagua..	1		1	Fm3
....................aaggauguuugacaaguU..	5		1	Fm3
....................aaggauguuugacaaguG..	2		1	Fm3
....................aaggauguuugacaagGa..	2		1	Fm3
....................aaggCuguuugacaagua..	1		1	Fm3
....................aaggauguuGgacaagua..	1		1	Fm3
....................aaggauguuugacaagua..	284		0	Fm3
....................aaggauguuugacaagAa..	1		1	Fm3
....................aaggauguuugacaagCag...	2		1	Fm3
....................aaggauguuugacaaguGg...	1		1	Fm3
....................aaggauguuugacaaguaU...	15		1	Fm3
....................aaggauguuugacaaguaA...	39		1	Fm3
....................aaggauguuugaUaaguag...	1		1	Fm3
....................aaAgauguuugacaaguag...	1		1	Fm3
....................aaggauguuuAacaaguag...	2		1	Fm3
....................aaggauguuugacaaguCg...	1		1	Fm3
....................aaggauguuugacaaguag...	175		0	Fm3
....................aaggaugCuugacaaguagu..	3		1	Fm3
....................aaggauguuugacaaguUgu..	13		1	Fm3
....................aaggauguuugacaagAagu..	3		1	Fm3
....................aaggauguuugaGaaguagu..	2		1	Fm3
....................aCggauguuugacaaguagu..	1		1	Fm3
....................Uaggauguuugacaaguagu..	7		1	Fm3
....................aaggauguuugGcaaguagu..	1		1	Fm3
....................aaggauUuuugacaaguagu..	1		1	Fm3

Mature Star

```
aaccuugguauaugaaacguaaggauguuugacaaguaguuugugcgucgacaaacugcuuuuugaacauacuccucguucuuuaccgagucugcuuauugcuucacgug
....................aaggauguuugacaaguCgu......................................................................   15      1    Fm3
....................aUggauguuugacaaguagu......................................................................   1       1    Fm3
....................aGggauguuugacaaguagu......................................................................   6       1    Fm3
....................aaggauguuCgacaaguagu......................................................................   1       1    Fm3
....................aagAauguuugacaaguagu......................................................................   1       1    Fm3
....................aaggaAguuugacaaguagu......................................................................   2       1    Fm3
....................aaggauguuugacaagCagu......................................................................   15      1    Fm3
....................aaUgauguuugacaaguagu......................................................................   6       1    Fm3
....................aaggauguuugacaaguGgu......................................................................   13      1    Fm3
....................aaggauCuuugacaaguagu......................................................................   1       1    Fm3
....................aaggCuguuugacaaguagu......................................................................   1       1    Fm3
....................aaggauguuugacaagGagu......................................................................   17      1    Fm3
....................aaggauguuugacGaguagu......................................................................   1       1    Fm3
....................aaggauguuuUacaaguagu......................................................................   2       1    Fm3
....................aaggauguuuCacaaguagu......................................................................   1       1    Fm3
....................aaggauguuugacaaguaUu......................................................................   1       1    Fm3
....................aaggGuguuugacaaguagu......................................................................   2       1    Fm3
....................aaggauguuugacaaguaAu......................................................................   1       1    Fm3
....................aaggauguuugUcaaguagu......................................................................   4       1    Fm3
....................Naggauguuugacaaguagu......................................................................   2       1    Fm3
....................aaggauguuuAacaaguagu......................................................................   7       1    Fm3
....................aaggaugAuugacaaguagu......................................................................   1       1    Fm3
....................aagCauguuugacaaguagu......................................................................   1       1    Fm3
....................aaggauguuugacaaguagu......................................................................   3999    0    Fm3
....................aaggauguuugacaaguagA......................................................................   15      1    Fm3
....................Gaggauguuugacaaguagu......................................................................   5       1    Fm3
....................aaCgauguuugacaaguaguu.....................................................................   1       1    Fm3
....................aaggauguuuAacaaguaguu.....................................................................   23      1    Fm3
....................aaggauguuugacaagGaguu.....................................................................   46      1    Fm3
....................Gaggauguuugacaaguaguu.....................................................................   7       1    Fm3
....................aaggauguuugacaaguagCu.....................................................................   2       1    Fm3
....................aaggauguuugacUaguaguu.....................................................................   4       1    Fm3
....................aaAgauguuugacaaguaguu.....................................................................   2       1    Fm3
....................aaggauguGugacaaguaguu.....................................................................   1       1    Fm3
....................aaggauCuuugacaaguaguu.....................................................................   2       1    Fm3
....................aaggauguuugacaagCaguu.....................................................................   55      1    Fm3
....................aaggauNuuugacaaguaguu.....................................................................   1       1    Fm3
....................aaggaNguuugacaaguaguu.....................................................................   2       1    Fm3
....................aaggauguuugacaaguaUuu.....................................................................   1       1    Fm3
....................aaggauguuGgacaaguaguu.....................................................................   1       1    Fm3
....................aaggauAuuugacaaguaguu.....................................................................   1       1    Fm3
....................aaggauguuugacaaguaguC.....................................................................   2       1    Fm3
....................aaggauguuCgacaaguaguu.....................................................................   2       1    Fm3
....................aaggauguuugacaaguaguu.....................................................................   14292   0    Fm3
....................aagUauguuugacaaguaguu.....................................................................   3       1    Fm3
....................Caggauguuugacaaguaguu.....................................................................   1       1    Fm3
....................aaggauguuugacaaguaAuu.....................................................................   3       1    Fm3
....................aagAauguuugacaaguaguu.....................................................................   2       1    Fm3
....................aUggauguuugacaaguaguu.....................................................................   2       1    Fm3
....................aaggauguuugacaaguaguA.....................................................................   65      1    Fm3
....................aaggCuguuugacaaguaguu.....................................................................   5       1    Fm3
....................aaggauguuugacaGguaguu.....................................................................   2       1    Fm3
....................aaggauguuugacaaguagAu.....................................................................   1       1    Fm3
....................aaggauguuugacCaguaguu.....................................................................   1       1    Fm3
....................aaggauguuugacaaguUguu.....................................................................   22      1    Fm3
....................aaggGuguuugacaaguaguu.....................................................................   1       1    Fm3
....................aagCauguuugacaaguaguu.....................................................................   1       1    Fm3
....................aCggauguuugacaaguaguu.....................................................................   1       1    Fm3
....................aaggauguuugaGaaguaguu.....................................................................   1       1    Fm3
....................Naggauguuugacaaguaguu.....................................................................   3       1    Fm3
....................aaggauguuugacaaAuaguu.....................................................................   1       1    Fm3
....................aaUgauguuugacaaguaguu.....................................................................   6       1    Fm3
....................aaggauguuugacaUguaguu.....................................................................   3       1    Fm3
....................aaggaugCuugacaaguaguu.....................................................................   2       1    Fm3
....................aaggauguuugacaagAaguu.....................................................................   39      1    Fm3
....................aaggauguuuUacaaguaguu.....................................................................   12      1    Fm3
....................aaggUuguuugacaaguaguu.....................................................................   2       1    Fm3
....................aaggauguuugacaaguGguu.....................................................................   39      1    Fm3
....................aGggauguuugacaaguaguu.....................................................................   11      1    Fm3
....................aaggauguuugaAaaguaguu.....................................................................   2       1    Fm3
```

```
                    Mature                   Star
aaccuugguauaugaaacguaaggauguuugacaaguaguuuugugcgucgacaaacugcuuuuugaacauacuccucguuucuuuaccgagucugcuuauugcuucacgug
....................Uaggauguuugacaaguaguu.......................................................................   21      1   Fm3
....................aaggauguuuCacaaguaguu.......................................................................   9       1   Fm3
....................aaggauguuAgacaaguaguu.......................................................................   3       1   Fm3
....................aaggauguuugUcaaguaguu.......................................................................   7       1   Fm3
....................aaggauguuugacGaguaguu.......................................................................   2       1   Fm3
....................aaggauguuugacaCguaguu.......................................................................   1       1   Fm3
....................aaggauguuugaUaaguaguu.......................................................................   9       1   Fm3
....................aaggaugAuugacaaguaguu.......................................................................   1       1   Fm3
....................aaggauguuugacaaguCguu.......................................................................   68      1   Fm3
....................aaggauguAugacaaguaguu.......................................................................   2       1   Fm3
....................aaggauNuuugacaaguaguuu......................................................................   4       1   Fm3
....................aagUauguuugacaaguaguuu......................................................................   3       1   Fm3
....................aaggauguuugacaaguaguAu......................................................................   5       1   Fm3
....................aaggauguuAgacaaguaguuu......................................................................   2       1   Fm3
....................aaggauguuugacaGguaguuu......................................................................   4       1   Fm3
....................aaggauguuugacaaguaUuuu......................................................................   1       1   Fm3
....................aaggauguuugacaaUuaguuu......................................................................   11      1   Fm3
....................aaggauguuugacaaguGguuu......................................................................   132     1   Fm3
....................aaggauguuugacaagCaguuu......................................................................   160     1   Fm3
....................Gaggauguuugacaaguaguuu......................................................................   22      1   Fm3
....................aaggauAuuugacaaguaguuu......................................................................   4       1   Fm3
....................aaggauguuugacaaCuaguuu......................................................................   1       1   Fm3
....................aaggauguuuCacaaguaguuu......................................................................   55      1   Fm3
....................aaNgauguuugacaaguaguuu......................................................................   1       1   Fm3
....................aaggauguuugacaaAuaguuu......................................................................   4       1   Fm3
....................aaggauguuugacaagGaguuu......................................................................   119     1   Fm3
....................aaggauguuugacaaguaguuA......................................................................   398     1   Fm3
....................Naggauguuugacaaguaguuu......................................................................   27      1   Fm3
....................aagAauguuugacaaguaguuu......................................................................   3       1   Fm3
....................aaggauguuugacaaguaAuuu......................................................................   1       1   Fm3
....................aUggauguuugacaaguaguuu......................................................................   11      1   Fm3
....................aaggaugCuugacaaguaguuu......................................................................   8       1   Fm3
....................aaggauguuugaGaaguaguuu......................................................................   4       1   Fm3
....................aaggaNguuugacaaguaguuu......................................................................   3       1   Fm3
....................aaggauguuugacaaguUguuu......................................................................   105     1   Fm3
....................aaggauguuuUacaaguaguuu......................................................................   32      1   Fm3
....................aGggauguuugacaaguaguuu......................................................................   82      1   Fm3
....................aaggauguGugacaaguaguuu......................................................................   3       1   Fm3
....................aaggauguuugacaagAaguuu......................................................................   103     1   Fm3
....................aaggauguuugacaaguagGuu......................................................................   3       1   Fm3
....................aaggauguuugacaaguaguuu......................................................................   47728   0   Fm3
....................aaggauguuuAacaaguaguuu......................................................................   93      1   Fm3
....................aCggauguuugacaaguaguuu......................................................................   7       1   Fm3
....................aaggauguuugaAaaguaguuu......................................................................   1       1   Fm3
....................aaggauUuuugacaaguaguuu......................................................................   2       1   Fm3
....................aaggauguuugacaaguagAuu......................................................................   1       1   Fm3
....................aaggauguuugacaaguCguuu......................................................................   173     1   Fm3
....................aaggauguuugacUaguaguuu......................................................................   10      1   Fm3
....................aaggauguuugacaUguaguuu......................................................................   6       1   Fm3
....................aaggauguuugacGaguaguuu......................................................................   15      1   Fm3
....................aaggaAguuugacaaguaguuu......................................................................   1       1   Fm3
....................aaAgauguuugacaaguaguuu......................................................................   12      1   Fm3
....................aaggauguuugacCaguaguuu......................................................................   1       1   Fm3
....................aaUgauguuugacaaguaguuu......................................................................   15      1   Fm3
....................Uaggauguuugacaaguaguuu......................................................................   99      1   Fm3
....................aaggauguuugUcaaguaguuu......................................................................   9       1   Fm3
....................aaggUuguuugacaaguaguuu......................................................................   4       1   Fm3
....................aaggauguuugacaaguaguGu......................................................................   4       1   Fm3
....................aaggauguCugacaaguaguuu......................................................................   3       1   Fm3
....................aaggauguuugaUaaguaguuu......................................................................   11      1   Fm3
....................aaggaugGuugacaaguaguuu......................................................................   3       1   Fm3
....................aagCauguuugacaaguaguuu......................................................................   1       1   Fm3
....................aaggaugAuugacaaguaguuu......................................................................   2       1   Fm3
....................aaggauguuugacaaguaguCu......................................................................   1       1   Fm3
....................aaggauguuugacaaguaguuG......................................................................   1       1   Fm3
....................Caggauguuugacaaguaguuu......................................................................   7       1   Fm3
....................aaggauguuCgacaaguaguuu......................................................................   7       1   Fm3
....................aaggauguuugacaaguagCuu......................................................................   5       1   Fm3
....................aaggauguuugacaaguaguuC......................................................................   14      1   Fm3
....................aaggauguuugGcaaguaguuu......................................................................   4       1   Fm3
```

```
                    Mature                            Star
aaccuugguauaugaaacguaaggauguuugacaaguaguuuugugcgucgacaaacugcuuuuugaacauacuccucguucuuuaccgagucugcuuauugcuucacgug
....................aaggauguuGgacaaguaguuu.....................................................................   9     1    Fm3
....................aaggauguAugacaaguaguuu.....................................................................   5     1    Fm3
....................aaggCuguuugacaaguaguuu.....................................................................   11    1    Fm3
....................aaggGuguuugacaaguaguuu.....................................................................   15    1    Fm3
....................aaCgauguuugacaaguaguuu.....................................................................   1     1    Fm3
....................aaggaCguuugacaaguaguuu.....................................................................   3     1    Fm3
....................aaggauguuugacaaguaguuuA....................................................................   66    1    Fm3
....................aaggauguuugacaaguaguuAu....................................................................   17    1    Fm3
....................aaggauguuugacaaguUguuuu....................................................................   1     1    Fm3
....................aaggauguuugacaaguaguuCu....................................................................   1     1    Fm3
....................aaggauguuugacaaguCguuuu....................................................................   1     1    Fm3
....................aaggauguuugacaagAaguuuu....................................................................   1     1    Fm3
....................aaggauguuugacaaguaguuuu....................................................................   459   0    Fm3
....................aaggauguuuCacaaguaguuuu....................................................................   1     1    Fm3
....................aaggauguuugacaaguagGuuu....................................................................   1     1    Fm3
....................aaggauguuugacaaguGguuuu....................................................................   2     1    Fm3
....................aaggauguuuAacaaguaguuuu....................................................................   2     1    Fm3
....................aaggauguuugacaaguaguuuG....................................................................   1     1    Fm3
....................aaggauguuugacaagCaguuuu....................................................................   2     1    Fm3
....................aaggauguuugacaaguaguuGu....................................................................   4     1    Fm3
....................aaggCuguuugacaaguaguuuu....................................................................   1     1    Fm3
....................Uaggauguuugacaaguaguuuu....................................................................   3     1    Fm3
....................aaggauguuugacaaguaguuuuU...................................................................   2     1    Fm3
....................aaggauguuugacaaguaguuuuA...................................................................   1     1    Fm3
.....................aggauguuugacaaguaguu......................................................................   2     0    Fm3
.....................aggauguuugacaaguagGuu.....................................................................   1     1    Fm3
.....................aggauAuuugacaaguaguuu.....................................................................   1     1    Fm3
.....................aggauguuugacaaguaguuu.....................................................................   68    0    Fm3
.....................aggauguAugacaaguaguuu.....................................................................   1     1    Fm3
.....................aggauguuuUacaaguaguuu.....................................................................   1     1    Fm3
.....................aggauguuugacaaguCguuu.....................................................................   1     1    Fm3
.......................gauguuugacaaguaguuu.....................................................................   8     0    Fm3
.......................................................acugcuuuuugaacauacuccuc.................................   2     0    Fm3

...................uaaggauguuugacaaguagu.......................................................................   1     0    Mm3
...................uaaggauguuugacaaguaguu......................................................................   1     0    Mm3
...................Caaggauguuugacaaguaguuu.....................................................................   1     1    Mm3
...................Caaggauguuugacaaguaguuuu....................................................................   1     1    Mm3
....................aaggauguuugacaaguU.........................................................................   1     1    Mm3
....................aaggauguuugacaagua.........................................................................   13    0    Mm3
....................aaggauguuugacaaguag........................................................................   6     0    Mm3
....................aaggauguuugacaaguagu.......................................................................   196   0    Mm3
....................aaggauguuugacaaguCgu.......................................................................   1     1    Mm3
....................aaggauguuugacaagCagu.......................................................................   3     1    Mm3
....................aaggauguuugacaaguagA.......................................................................   1     1    Mm3
....................aGggauguuugacaaguagu.......................................................................   1     1    Mm3
....................Uaggauguuugacaaguagu.......................................................................   1     1    Mm3
....................aaggauguuugacaaguUgu.......................................................................   1     1    Mm3
....................aaggauguuugacaaguUguu......................................................................   3     1    Mm3
....................aaggauguuugacaaguGguu......................................................................   1     1    Mm3
....................aaggauguuugacaaguCguu......................................................................   3     1    Mm3
....................aCggauguuugacaaguaguu......................................................................   1     1    Mm3
....................aGggauguuugacaaguaguu......................................................................   1     1    Mm3
....................aaggauguuugacaaguaguu......................................................................   933   0    Mm3
....................aaggauguuugacaaguaguA......................................................................   3     1    Mm3
....................aaggauguuuUacaaguaguu......................................................................   1     1    Mm3
....................aaggauguuuCacaaguaguu......................................................................   1     1    Mm3
....................aaggGuguuugacaaguaguu......................................................................   1     1    Mm3
....................aaggauguuugacaagAaguu......................................................................   1     1    Mm3
....................aaggauguuugacaagCaguu......................................................................   2     1    Mm3
....................aaggauguuAgacaaguaguu......................................................................   1     1    Mm3
....................Uaggauguuugacaaguaguu......................................................................   1     1    Mm3
....................Gaggauguuugacaaguaguu......................................................................   2     1    Mm3
....................aaggauguuugacUaguaguu......................................................................   1     1    Mm3
....................aaggauguuugaUaaguaguuu.....................................................................   1     1    Mm3
....................aaggauguuuUacaaguaguuu.....................................................................   7     1    Mm3
....................aaggauguuugacaagGaguuu.....................................................................   21    1    Mm3
....................Uaggauguuugacaaguaguuu.....................................................................   24    1    Mm3
....................aaggauUuuugacaaguaguuu.....................................................................   2     1    Mm3
....................aUggauguuugacaaguaguuu.....................................................................   6     1    Mm3
```

```
                    Mature                             Star
aaccuugguauaugaaacguaaggauguuugacaaguaguuuugugcgucgacaaacugcuuuuugaacauacuccucguucuuuaccgagucugcuuauugcuucacgug
....................aaggauguuugacUaguaguuu.....................................................................   1      1   Mm3
....................aaggGuguuugacaaguaguuu.....................................................................   3      1   Mm3
....................aaggauguuugacaaguaguuA.....................................................................   19     1   Mm3
....................aaggauguuugacaGguaguuu.....................................................................   1      1   Mm3
....................aaggauguuugUcaaguaguuu.....................................................................   1      1   Mm3
....................aaggauguAugacaaguaguuu.....................................................................   1      1   Mm3
....................aaggUuguuugacaaguaguuu.....................................................................   2      1   Mm3
....................aaggauguuuCacaaguaguuu.....................................................................   3      1   Mm3
....................Caggauguuugacaaguaguuu.....................................................................   1      1   Mm3
....................aaggauguuugacaagCaguuu.....................................................................   26     1   Mm3
....................aaggauguuugacaaguCguuu.....................................................................   29     1   Mm3
....................aaggauguuugCcaaguaguuu.....................................................................   1      1   Mm3
....................aaggauguuugacaCguaguuu.....................................................................   1      1   Mm3
....................Naggauguuugacaaguaguuu.....................................................................   3      1   Mm3
....................aaggauguuugacaaguaguAu.....................................................................   1      1   Mm3
....................aaggauguuugGcaaguaguuu.....................................................................   1      1   Mm3
....................aNggauguuugacaaguaguuu.....................................................................   1      1   Mm3
....................Gaggauguuugacaaguaguuu.....................................................................   11     1   Mm3
....................aaggauguuugacGaguaguuu.....................................................................   3      1   Mm3
....................aaggauguCugacaaguaguuu.....................................................................   1      1   Mm3
....................aaggauguuGgacaaguaguuu.....................................................................   1      1   Mm3
....................aaggauguuugacaaguaguuC.....................................................................   1      1   Mm3
....................aaggauguuugacaaguaguuu.....................................................................   7057   0   Mm3
....................aaggauguuugacaaguGguuu.....................................................................   14     1   Mm3
....................aaggCuguuugacaaguaguuu.....................................................................   6      1   Mm3
....................aaggauguuugacaaUuaguuu.....................................................................   1      1   Mm3
....................aaggauguuuAacaaguaguuu.....................................................................   9      1   Mm3
....................aaggaugCuugacaaguaguuu.....................................................................   1      1   Mm3
....................aaggauguuugacaaguaguuG.....................................................................   1      1   Mm3
....................aaggauguuugacaaguUguuu.....................................................................   23     1   Mm3
....................aaCgauguuugacaaguaguuu.....................................................................   2      1   Mm3
....................aaggaCguuugacaaguaguuu.....................................................................   3      1   Mm3
....................aaAgauguuugacaaguaguuu.....................................................................   2      1   Mm3
....................aCggauguuugacaaguaguuu.....................................................................   1      1   Mm3
....................aaggauguuugacCaguaguuu.....................................................................   1      1   Mm3
....................aGggauguuugacaaguaguuu.....................................................................   21     1   Mm3
....................aaggauguuugacaagAaguuu.....................................................................   10     1   Mm3
....................aaggauguuugacaaguagCuu.....................................................................   1      1   Mm3
....................Gaggauguuugacaaguaguuuu....................................................................   1      1   Mm3
....................aaggUuguuugacaaguaguuuu....................................................................   2      1   Mm3
....................aaggauguuugacaaUuaguuuu....................................................................   1      1   Mm3
....................aaAgauguuugacaaguaguuuu....................................................................   1      1   Mm3
....................aaggauguuugacaagAaguuuu....................................................................   3      1   Mm3
....................aaggauguuugacaaguaguuuA....................................................................   23     1   Mm3
....................aaggauguuugacaaguaguuuu....................................................................   481    0   Mm3
....................aaggauguuugacaaguUguuuu....................................................................   1      1   Mm3
....................aaggauguuugacaagCaguuuu....................................................................   2      1   Mm3
....................aaggauguuugUcaaguaguuuu....................................................................   1      1   Mm3
....................Uaggauguuugacaaguaguuuu....................................................................   1      1   Mm3
....................aGggauguuugacaaguaguuuu....................................................................   2      1   Mm3
....................aaggauguuugacaaguaguuAu....................................................................   2      1   Mm3
....................aaggauguuugacaagGaguuuu....................................................................   1      1   Mm3
....................aaggauguuugacaaguaguuuuU...................................................................   20     1   Mm3
....................aaggauguuugacaaguaguuuuUu..................................................................   8      1   Mm3
.....................aggauguuugacaaguaguu......................................................................   1      0   Mm3
.....................aggauguuugacaaguaguuu.....................................................................   10     0   Mm3
.....................aUgauguuugacaaguaguuu.....................................................................   1      1   Mm3
.....................aggauguuugacaaguaguuuu....................................................................   1      0   Mm3
.......................Aauguuugacaaguaguuu.....................................................................   1      1   Mm3
.......................................................acugcuuuuugaacauacuccu..................................   4      0   Mm3
.......................................................acugcuuuuugaacauacuccuc.................................   5      0   Mm3

...................Caaggauguuugacaaguaguuu.....................................................................   13     1   Fm2
....................aaggauguuugacaagua.........................................................................   31     0   Fm2
....................aGggauguuugacaaguag........................................................................   1      1   Fm2
....................aaggauguuugacaaguag........................................................................   12     0   Fm2
....................aaggauguuugacaaguGgu.......................................................................   1      1   Fm2
....................aaggauguAugacaaguagu.......................................................................   1      1   Fm2
....................aaggauguuugGcaaguagu.......................................................................   1      1   Fm2
....................aaCgauguuugacaaguagu.......................................................................   1      1   Fm2
```

Mature **Star**

aaccuugguauaugaaacguaaggauguuugacaaguaguuugugcgucgacaaacugcuuuuugaacauacuccucguuucuuuaccgagucugcuuauugcuucacgug			
....................aaggauguuuAacaaguagu...	3	1	Fm2
....................aaggauguuuCacaaguagu...	1	1	Fm2
....................aaggauguuugacaaguagu...	858	0	Fm2
....................aaggauguuugacaaguagA...	7	1	Fm2
....................aaggauguuugacaaguagG...	2	1	Fm2
....................aaggauguuCgacaaguagu...	1	1	Fm2
....................aaggauguuugacaaguUgu...	4	1	Fm2
....................aaggauguuugacaagAagu...	3	1	Fm2
....................aaggauguuugacaaguCgu...	2	1	Fm2
....................aaggauguuugacaagCagu...	5	1	Fm2
....................Uaggauguuugacaaguagu...	2	1	Fm2
....................aUggauguuugacaaguaguu..	1	1	Fm2
....................aaggauguuugacaagCaguu..	11	1	Fm2
....................aaggauguuugacaaguUguu..	11	1	Fm2
....................aaggaugGuugacaaguaguu..	1	1	Fm2
....................aaggauguuuUacaaguaguu..	2	1	Fm2
....................Gaggauguuugacaaguaguu..	8	1	Fm2
....................aaggauUuuugacaaguaguu..	1	1	Fm2
....................aaggauguuugacaagAaguu..	11	1	Fm2
....................aaggaNguuugacaaguaguu..	3	1	Fm2
....................aaggauguuugacaaguaguG..	2	1	Fm2
....................aaggCuguuugacaaguaguu..	3	1	Fm2
....................aaUgauguuugacaaguaguu..	1	1	Fm2
....................Naggauguuugacaaguaguu..	1	1	Fm2
....................aaggauguuugaUaaguaguu..	1	1	Fm2
....................aaggauguuugaGaaguaguu..	1	1	Fm2
....................Uaggauguuugacaaguaguu..	4	1	Fm2
....................aGggauguuugacaaguaguu..	9	1	Fm2
....................aaggauguuugUcaaguaguu..	1	1	Fm2
....................aaggauguuugacaaguaguA..	14	1	Fm2
....................aagCauguuugacaaguaguu..	1	1	Fm2
....................aaggauguuugacaaguaguu..	3752	0	Fm2
....................aCggauguuugacaaguaguu..	1	1	Fm2
....................aaggauguuugacGaguaguu..	1	1	Fm2
....................aaggauguuugacaaguCguu..	19	1	Fm2
....................aaggGuguuugacaaguaguu..	3	1	Fm2
....................aaggauguuugacaagGaguu..	11	1	Fm2
....................aaggauguuugacaaguGguu..	10	1	Fm2
....................aaggauguuugacaUguaguu..	1	1	Fm2
....................aaggauguuuCacaaguaguu..	2	1	Fm2
....................aaggauguuuAacaaguaguu..	4	1	Fm2
....................Caggauguuugacaaguaguuu...	3	1	Fm2
....................aUggauguuugacaaguaguuu...	16	1	Fm2
....................aaggauguuuAacaaguaguuu...	66	1	Fm2
....................aaAgauguuugacaaguaguuu...	5	1	Fm2
....................aaggauguuugacaaguagAuu...	4	1	Fm2
....................aaggauguuugacaaguCguuu...	144	1	Fm2
....................aaggauguuugUcaaguaguuu...	21	1	Fm2
....................aaggauguuugacaCguaguuu...	1	1	Fm2
....................aaggauguuugacaaguaguuC...	1	1	Fm2
....................aaggauUuuugacaaguaguuu...	6	1	Fm2
....................aaggauguuugacaaguaguAu...	2	1	Fm2
....................aaggauguuugacaaguagGuu...	1	1	Fm2
....................aaggauguuAgacaaguaguuu...	5	1	Fm2
....................aaggauguCugacaaguaguuu...	6	1	Fm2
....................aaggauguuugacaagAaguuu...	80	1	Fm2
....................aaggauAuuugacaaguaguuu...	5	1	Fm2
....................aaggauguuGgacaaguaguuu...	2	1	Fm2
....................aaggauguuugacaaguNguuu...	1	1	Fm2
....................aaggauguuugacCaguaguuu...	4	1	Fm2
....................aaggauguuugacaaguagCuu...	1	1	Fm2
....................aaggauguuugacaaguaguGu...	2	1	Fm2
....................Naggauguuugacaaguaguuu...	18	1	Fm2
....................aaggauguuugacaagGaguuu...	90	1	Fm2
....................aaUgauguuugacaaguaguuu...	11	1	Fm2
....................aaggauguuugacaagCaguuu...	143	1	Fm2
....................aaggauguuugacaaguaguCu...	3	1	Fm2
....................aaggauguuugacaaguaguuG...	21	1	Fm2
....................aaggaCguuugacaaguaguuu...	5	1	Fm2
....................Uaggauguuugacaaguaguuu...	69	1	Fm2

```
                    Mature                         Star
aaccuugguauaugaaacguaaggauguuugacaaguaguuuugugcgucgacaaacugcuuuuugaacauacuccucguuucuuuaccgagucugcuuauugcuucacgug
....................aaggauguuugacaaguaguuu......................................................................    38348   0   Fm2
....................aaggUuguuugacaaguaguuu......................................................................    12      1   Fm2
....................aaggauguuugCcaaguaguuu......................................................................    1       1   Fm2
....................aaggauguuugacaaUuaguuu......................................................................    1       1   Fm2
....................aaggGuguuugacaaguaguuu......................................................................    10      1   Fm2
....................aaggauNuuugacaaguaguuu......................................................................    1       1   Fm2
....................aaggauguuugacaaguaguuA......................................................................    153     1   Fm2
....................aaggauguuugacGaguaguuu......................................................................    3       1   Fm2
....................aaggauguuugacaaAuaguuu......................................................................    2       1   Fm2
....................aaggaugCuugacaaguaguuu......................................................................    6       1   Fm2
....................aaggauguuuCacaaguaguuu......................................................................    24      1   Fm2
....................aaNgauguuugacaaguaguuu......................................................................    1       1   Fm2
....................aaggauguuugacaUguaguuu......................................................................    7       1   Fm2
....................aaggauguAugacaaguaguuu......................................................................    7       1   Fm2
....................aaggauguGugacaaguaguuu......................................................................    3       1   Fm2
....................aaggauguuuUacaaguaguuu......................................................................    39      1   Fm2
....................aCggauguuugacaaguaguuu......................................................................    6       1   Fm2
....................aagAauguuugacaaguaguuu......................................................................    2       1   Fm2
....................aaggaugGuugacaaguaguuu......................................................................    1       1   Fm2
....................aaggauCuuugacaaguaguuu......................................................................    1       1   Fm2
....................aaggauguuugacaaguUguuu......................................................................    75      1   Fm2
....................aaggauguuCgacaaguaguuu......................................................................    4       1   Fm2
....................aaggauguuugaUaaguaguuu......................................................................    9       1   Fm2
....................aaggauguuugacaaguGguuu......................................................................    109     1   Fm2
....................aaggauguuugacaaguaUuuu......................................................................    3       1   Fm2
....................aaggaugAuugacaaguaguuu......................................................................    3       1   Fm2
....................aagUauguuugacaaguaguuu......................................................................    2       1   Fm2
....................aaggauguuugacaGguaguuu......................................................................    8       1   Fm2
....................aaggauguuugacaaCuaguuu......................................................................    1       1   Fm2
....................aaggaAguuugacaaguaguuu......................................................................    5       1   Fm2
....................aaggCuguuugacaaguaguuu......................................................................    17      1   Fm2
....................aaggaNguuugacaaguaguuu......................................................................    3       1   Fm2
....................aaggauguuugacUaguaguuu......................................................................    5       1   Fm2
....................aGggauguuugacaaguaguuu......................................................................    148     1   Fm2
....................aagCauguuugacaaguaguuu......................................................................    3       1   Fm2
....................aaggauguuugGcaaguaguuu......................................................................    2       1   Fm2
....................aaCgauguuugacaaguaguuu......................................................................    3       1   Fm2
....................Gaggauguuugacaaguaguuu......................................................................    102     1   Fm2
....................aNggauguuugacaaguaguuu......................................................................    1       1   Fm2
....................aaggaugGuugacaaguaguuuu.....................................................................    1       1   Fm2
....................aaggauguuugacaagAaguuuu.....................................................................    8       1   Fm2
....................aaggauguuugacaagGaguuuu.....................................................................    8       1   Fm2
....................aaggauguuugacGaguaguuuu.....................................................................    1       1   Fm2
....................aaggauAuuugacaaguaguuuu.....................................................................    1       1   Fm2
....................aaggauguuugacaaguaguGuu.....................................................................    1       1   Fm2
....................aaggauguuugacaaguagGuuu.....................................................................    1       1   Fm2
....................aaggauguuuUacaaguaguuuu.....................................................................    2       1   Fm2
....................aaggauguuugUcaaguaguuuu.....................................................................    2       1   Fm2
....................aaggauguuugaUaaguaguuuu.....................................................................    7       1   Fm2
....................aGggauguuugacaaguaguuuu.....................................................................    16      1   Fm2
....................aaggCuguuugacaaguaguuuu.....................................................................    3       1   Fm2
....................aaggauguuugacaaguaguuuG.....................................................................    1       1   Fm2
....................aaggauguuugacaaguCguuuu.....................................................................    11      1   Fm2
....................aaggauguuugacaaguaguuGu.....................................................................    5       1   Fm2
....................aaggaAguuugacaaguaguuuu.....................................................................    1       1   Fm2
....................aaggauguuugacaaguagAuuu.....................................................................    1       1   Fm2
....................aaggauguuuCacaaguaguuuu.....................................................................    7       1   Fm2
....................aaggauguuugacaaguaUuuuu.....................................................................    1       1   Fm2
....................aaggauguuugacaaguUguuuu.....................................................................    12      1   Fm2
....................aaggauguuugacaaguaguuCu.....................................................................    1       1   Fm2
....................aaggauguuugacaaguaguuuA.....................................................................    210     1   Fm2
....................Gaggauguuugacaaguaguuuu.....................................................................    14      1   Fm2
....................aaggauguuugacaaguGguuuu.....................................................................    8       1   Fm2
....................Naggauguuugacaaguaguuuu.....................................................................    4       1   Fm2
....................aaggauguuugacaagCaguuuu.....................................................................    11      1   Fm2
....................aaggauguuGgacaaguaguuuu.....................................................................    1       1   Fm2
....................aaggauguCugacaaguaguuuu.....................................................................    1       1   Fm2
....................aaggauguuugacaaguaAuuuu.....................................................................    1       1   Fm2
....................aaggauguuugacaaguaguuAu.....................................................................    15      1   Fm2
....................aaggauguuugacaaguaguuuu.....................................................................    3887    0   Fm2
```

```
                    Mature                             Star
aaccuugguauaugaaacguaaggauguuugacaaguaguuuugugcgucgacaaacugcuuuuugaacauacuccucguuucuuuaccgagucugcuuauugcuucacgug
....................aaggauguuAgacaaguaguuuu.....................................................................   2      1    Fm2
....................aaggauguuugacCaguaguuuu.....................................................................   1      1    Fm2
....................aaggauguAugacaaguaguuuu.....................................................................   1      1    Fm2
....................aaggauguuuAacaaguaguuuu.....................................................................   11     1    Fm2
....................aaUgauguuugacaaguaguuuu.....................................................................   2      1    Fm2
....................aaggaugAuugacaaguaguuuu.....................................................................   1      1    Fm2
....................aaggGuguuugacaaguaguuuu.....................................................................   2      1    Fm2
....................Uaggauguuugacaaguaguuuu.....................................................................   6      1    Fm2
....................aaggauguuugacaaguaguuuuU....................................................................   172    1    Fm2
....................aaggauguuugacaaguaguuuuUu...................................................................   19     1    Fm2
.....................aggauguuugacaaguaguu.......................................................................   1      0    Fm2
.....................aAgauguuugacaaguaguuu......................................................................   1      1    Fm2
.....................aggauguuugacaaguaguuu......................................................................   11     0    Fm2

...................Caaggauguuugacaaguagu........................................................................   3      1    Fm1
...................Caaggauguuugacaaguaguu.......................................................................   2      1    Fm1
...................uaaggauguuugacaaguaguu.......................................................................   3      0    Fm1
...................Caaggauguuugacaaguaguuu......................................................................   3      1    Fm1
...................Caaggauguuugacaaguaguuuu.....................................................................   2      1    Fm1
....................aaggauguuugacaagua..........................................................................   117    0    Fm1
....................aaggauguuugacaaguG..........................................................................   1      1    Fm1
....................aaggauguuugacaaguU..........................................................................   8      1    Fm1
....................aaggauguuugacaagCag.........................................................................   1      1    Fm1
....................aaggauguuugacaaguaU.........................................................................   4      1    Fm1
....................aaggauguuugacaaguaA.........................................................................   5      1    Fm1
....................aaggauguuugacaaguag.........................................................................   64     0    Fm1
....................aaggUuguuugacaaguagu........................................................................   1      1    Fm1
....................aaggauguuugaUaaguagu........................................................................   1      1    Fm1
....................Naggauguuugacaaguagu........................................................................   1      1    Fm1
....................aaggauguuugacaagAagu........................................................................   7      1    Fm1
....................aaggauguuuAacaaguagu........................................................................   1      1    Fm1
....................Uaggauguuugacaaguagu........................................................................   2      1    Fm1
....................Gaggauguuugacaaguagu........................................................................   2      1    Fm1
....................aaggauguuugacaaguagA........................................................................   13     1    Fm1
....................aaggauguuugacaagGagu........................................................................   2      1    Fm1
....................aaggauguuugacaaguagG........................................................................   2      1    Fm1
....................aaggauguuuCacaaguagu........................................................................   2      1    Fm1
....................aCggauguuugacaaguagu........................................................................   1      1    Fm1
....................aaggauguuugacaaguGgu........................................................................   3      1    Fm1
....................aaggauguuugacaagCagu........................................................................   6      1    Fm1
....................aaggauguuuUacaaguagu........................................................................   2      1    Fm1
....................aaggauguuugacUaguagu........................................................................   1      1    Fm1
....................aaggauguCugacaaguagu........................................................................   1      1    Fm1
....................aaggauguuugacaaguUgu........................................................................   3      1    Fm1
....................aaggauguuugacaaguaUu........................................................................   6      1    Fm1
....................aaggauguuugacaaguCgu........................................................................   3      1    Fm1
....................aaggauguuugacaaguagu........................................................................   1026   0    Fm1
....................aaggauguuugaUaaguaguu.......................................................................   1      1    Fm1
....................aaggaugCuugacaaguaguu.......................................................................   1      1    Fm1
....................aaggauguGugacaaguaguu.......................................................................   1      1    Fm1
....................aaggauguuugacaaguCguu.......................................................................   29     1    Fm1
....................aaggauguuugacUaguaguu.......................................................................   2      1    Fm1
....................aaggauguuCgacaaguaguu.......................................................................   1      1    Fm1
....................aaggauguuugacaagAaguu.......................................................................   17     1    Fm1
....................aaggauguuugacaaguaguG.......................................................................   8      1    Fm1
....................aagCauguuugacaaguaguu.......................................................................   1      1    Fm1
....................aaggauguuugacaaguaguC.......................................................................   1      1    Fm1
....................aaggGuguuugacaaguaguu.......................................................................   2      1    Fm1
....................aaggauguuugacCaguaguu.......................................................................   1      1    Fm1
....................aaggUuguuugacaaguaguu.......................................................................   1      1    Fm1
....................aaggauguuugacaaguaguu.......................................................................   5946   0    Fm1
....................aUggauguuugacaaguaguu.......................................................................   1      1    Fm1
....................aaggauguuugacaUguaguu.......................................................................   1      1    Fm1
....................aaggauguuugacaaguagCu.......................................................................   2      1    Fm1
....................aaggauguuuAacaaguaguu.......................................................................   11     1    Fm1
....................aaggauguAugacaaguaguu.......................................................................   1      1    Fm1
....................aaggauguuuCacaaguaguu.......................................................................   8      1    Fm1
....................aaggauguuugacaagCaguu.......................................................................   27     1    Fm1
....................aaggauguuugacaaguaguA.......................................................................   20     1    Fm1
....................aGggauguuugacaaguaguu.......................................................................   16     1    Fm1
```

```
                    Mature                 Star
aaccuugguauaugaaacguaaggauguuugacaaguaguuuugugcgucgacaaacugcuuuuugaacauacuccucguuucuuuaccgagucugcuuauugcuucacgug
....................aaggauguuugacaagGaguu.......................................................................  19     1  Fm1
....................aaggauguuugacaaguUguu.......................................................................  17     1  Fm1
....................aaggauguuugUcaaguaguu.......................................................................  2      1  Fm1
....................aaggauguuuUacaaguaguu.......................................................................  2      1  Fm1
....................Uaggauguuugacaaguaguu.......................................................................  13     1  Fm1
....................aaggauguuugacaaguagAu.......................................................................  1      1  Fm1
....................aaggauguuugacaaguGguu.......................................................................  17     1  Fm1
....................aaggauguuugacaCguaguu.......................................................................  1      1  Fm1
....................aaggaCguuugacaaguaguu.......................................................................  1      1  Fm1
....................Gaggauguuugacaaguaguu.......................................................................  8      1  Fm1
....................aaggauguuugacGaguaguu.......................................................................  1      1  Fm1
....................Naggauguuugacaaguaguu.......................................................................  1      1  Fm1
....................aaAgauguuugacaaguaguuu......................................................................  7      1  Fm1
....................aaggauguuugacGaguaguuu......................................................................  12     1  Fm1
....................aaggauguuugacaGguaguuu......................................................................  3      1  Fm1
....................aaggauguuugacaCguaguuu......................................................................  2      1  Fm1
....................aaggaNguuugacaaguaguuu......................................................................  10     1  Fm1
....................aaggauguuGgacaaguaguuu......................................................................  5      1  Fm1
....................aaggauguuugCcaaguaguuu......................................................................  2      1  Fm1
....................aaUgauguuugacaaguaguuu......................................................................  10     1  Fm1
....................aaggauguuugacaaguCguuu......................................................................  187    1  Fm1
....................aaCgauguuugacaaguaguuu......................................................................  2      1  Fm1
....................Caggauguuugacaaguaguuu......................................................................  3      1  Fm1
....................Naggauguuugacaaguaguuu......................................................................  20     1  Fm1
....................aaggauguuugacaaguUguuu......................................................................  140    1  Fm1
....................aaggCuguuugacaaguaguuu......................................................................  12     1  Fm1
....................aaggauguuugacUaguaguuu......................................................................  6      1  Fm1
....................aaggauguuugacaagGaguuu......................................................................  129    1  Fm1
....................aaggauguAugacaaguaguuu......................................................................  3      1  Fm1
....................aaggaGguuugacaaguaguuu......................................................................  2      1  Fm1
....................aaggauguuugacaaguagCuu......................................................................  2      1  Fm1
....................aaggauguuugacaagCaguuu......................................................................  145    1  Fm1
....................aaggUuguuugacaaguaguuu......................................................................  6      1  Fm1
....................aaggauguuugaAaaguaguuu......................................................................  3      1  Fm1
....................aaggauguuugacaaguaguCu......................................................................  3      1  Fm1
....................aaggauguuugacaaguaguuu......................................................................  47586  0  Fm1
....................aCggauguuugacaaguaguuu......................................................................  3      1  Fm1
....................aaggauguuugacaaguaguuG......................................................................  44     1  Fm1
....................aaggauguuugUcaaguaguuu......................................................................  11     1  Fm1
....................aaggauguuugacaaCuaguuu......................................................................  2      1  Fm1
....................aaggauguuugaUaaguaguuu......................................................................  16     1  Fm1
....................aaggaCguuugacaaguaguuu......................................................................  4      1  Fm1
....................aaggauguuugacaaguaUuuu......................................................................  4      1  Fm1
....................aNggauguuugacaaguaguuu......................................................................  1      1  Fm1
....................aaggauguuugacaaguGguuu......................................................................  130    1  Fm1
....................aagCauguuugacaaguaguuu......................................................................  2      1  Fm1
....................aaggaugGuugacaaguaguuu......................................................................  3      1  Fm1
....................aaggauguuugacaaguagAuu......................................................................  4      1  Fm1
....................aaggauguuuAacaaguaguuu......................................................................  74     1  Fm1
....................aaggauguuugacaaguagGuu......................................................................  6      1  Fm1
....................aaggGuguuugacaaguaguuu......................................................................  10     1  Fm1
....................aagAauguuugacaaguaguuu......................................................................  6      1  Fm1
....................aaggauguuugacaagAaguuu......................................................................  91     1  Fm1
....................aagUauguuugacaaguaguuu......................................................................  2      1  Fm1
....................aaggauguuugacaaguaguuC......................................................................  7      1  Fm1
....................aaggauguuuCacaaguaguuu......................................................................  38     1  Fm1
....................aaggauUuuugacaaguaguuu......................................................................  3      1  Fm1
....................aaggauguuugacaUguaguuu......................................................................  9      1  Fm1
....................aaggauguuugacaaguaguGu......................................................................  2      1  Fm1
....................aaggauguuCgacaaguaguuu......................................................................  7      1  Fm1
....................aUggauguuugacaaguaguuu......................................................................  14     1  Fm1
....................aaggaAguuugacaaguaguuu......................................................................  4      1  Fm1
....................aaggauguuugacCaguaguuu......................................................................  3      1  Fm1
....................aaggauguuugacaaguaguAu......................................................................  5      1  Fm1
....................aaggauguuugacaaguaguuA......................................................................  129    1  Fm1
....................aaggaugCuugacaaguaguuu......................................................................  3      1  Fm1
....................aaggauguGugacaaguaguuu......................................................................  2      1  Fm1
....................aaggauguuAgacaaguaguuu......................................................................  1      1  Fm1
....................aGggauguuugacaaguaguuu......................................................................  116    1  Fm1
....................aaggaugAuugacaaguaguuu......................................................................  5      1  Fm1
```

Mature Star

aaccuugguauaugaaacguaaggauguuugacaaguaguuuugugcgucgacaaacugcuuuuugaacauacuccucguuucuuuaccgagucugcuuauugcuucacgug			
....................aaggauguuugacaaAuaguuu..	2	1	Fm1
....................aaggauCuuugacaaguaguuu..	2	1	Fm1
....................aaggauAuuugacaaguaguuu..	2	1	Fm1
....................Uaggauguuugacaaguaguuu..	93	1	Fm1
....................aaggauNuuugacaaguaguuu..	2	1	Fm1
....................aaggauguuugGcaaguaguuu..	3	1	Fm1
....................aaNgauguuugacaaguaguuu..	1	1	Fm1
....................Gaggauguuugacaaguaguuu..	46	1	Fm1
....................aaggauguCugacaaguaguuu..	4	1	Fm1
....................aaggauguuuUacaaguaguuu..	34	1	Fm1
....................aaggauguuugacaaguaAuuu..	1	1	Fm1
....................aaggauguuuUacaaguaguuuu...	2	1	Fm1
....................aagCauguuugacaaguaguuuu...	1	1	Fm1
....................Naggauguuugacaaguaguuuu...	5	1	Fm1
....................aaggGuguuugacaaguaguuuu...	2	1	Fm1
....................aaggauguuugacaagGaguuuu...	3	1	Fm1
....................aaggauguuugacaaCuaguuuu...	1	1	Fm1
....................aaggaNguuugacaaguaguuuu...	1	1	Fm1
....................aaggauguuugUcaaguaguuuu...	2	1	Fm1
....................aaggauguuugacaagCaguuuu...	12	1	Fm1
....................aaggauUuuugacaaguaguuuu...	1	1	Fm1
....................aaggauguuugacaaguaguuuA...	100	1	Fm1
....................aaggauguuuAacaaguaguuuu...	4	1	Fm1
....................aGggauguuugacaaguaguuuu...	1	1	Fm1
....................Uaggauguuugacaaguaguuuu...	3	1	Fm1
....................aaggauguuugacaaguagGuuu...	2	1	Fm1
....................aaggauguuugacUaguaguuuu...	3	1	Fm1
....................aaggauguuugCcaaguaguuuu...	1	1	Fm1
....................aaggauguuCgacaaguaguuuu...	1	1	Fm1
....................aaAgauguuugacaaguaguuuu...	1	1	Fm1
....................aaggauguAugacaaguaguuuu...	2	1	Fm1
....................aaggCuguuugacaaguaguuuu...	1	1	Fm1
....................aaggauguuugacaaguUguuuu...	9	1	Fm1
....................aaggauguGugacaaguaguuuu...	1	1	Fm1
....................aaggauguuugacaUguaguuuu...	2	1	Fm1
....................aaggauguuuCacaaguaguuuu...	2	1	Fm1
....................Gaggauguuugacaaguaguuuu...	1	1	Fm1
....................aaggauguuugacaaguGguuuu...	14	1	Fm1
....................aaggauguuugacaaguaguuuC...	3	1	Fm1
....................aaggauguuugacaagAaguuuu...	9	1	Fm1
....................aaggauguuugacaaguaguuCu...	1	1	Fm1
....................aaggauguuugacaaguaguuAu...	6	1	Fm1
....................aaggauguuugacaaguaguuuG...	4	1	Fm1
....................aaggauguuugacaaguCguuuu...	17	1	Fm1
....................aaggauguuugacaaguaguuuu...	3791	0	Fm1
....................aaggauguuugacaaguaguuuuU..	139	1	Fm1
....................aaggauguuugacaaguaguuuuC..	3	1	Fm1
....................aaggauguuugacaaguaguuuuA..	1	1	Fm1
....................aaggauguuugacaaguaguuuuUu...	14	1	Fm1
.....................aggauguuugUcaaguaguuu..	2	1	Fm1
.....................aggauguuugacaaguaguuu..	55	0	Fm1
.....................aggaugGuugacaaguaguuu..	1	1	Fm1
.....................aggauguuugacaaguaguuuu...	1	0	Fm1
....acugcuuuuugaacauacuccuc..................................	21	0	Fm1
....acugcuuuuugaacauacuccucU.................................	3	1	Fm1
...................uaaggauguuugacaaguaguu...	2	0	Mm2
...................uaaggauguuugacaaguaguuu..	1	0	Mm2
...................Caaggauguuugacaaguaguuu..	17	1	Mm2
....................aaggauguuugacaagua..	22	0	Mm2
....................aaggauguuugacaaguU..	1	1	Mm2
....................aaggauguuugacaaguag...	13	0	Mm2
....................aaggauguuugacaaguUg...	1	1	Mm2
....................aaggauguuugacaagAagu..	1	1	Mm2
....................aaggauguuugacaaguagu..	445	0	Mm2
....................Gaggauguuugacaaguagu..	1	1	Mm2
....................aaggCuguuugacaaguagu..	1	1	Mm2
....................Uaggauguuugacaaguagu..	2	1	Mm2
....................aaggauguuugacaaguUgu..	1	1	Mm2
....................aGggauguuugacaaguagu..	2	1	Mm2

```
                    Mature                            Star
aaccuugguauaugaaacguaaggauguuugacaaguaguuuugugcgucgacaaacugcuuuuugaacauacuccucguuucuuuaccgagucugcuuauugcuucacgug
....................aaggauguuugacaaguGgu........................................................................   2       1   Mm2
....................aaggauguuugacaaguCgu........................................................................   1       1   Mm2
....................aaggauguuugUcaaguaguu.......................................................................   1       1   Mm2
....................aUggauguuugacaaguaguu.......................................................................   2       1   Mm2
....................aaggauguuugacUaguaguu.......................................................................   2       1   Mm2
....................aaggauguuuUacaaguaguu.......................................................................   3       1   Mm2
....................aGggauguuugacaaguaguu.......................................................................   18      1   Mm2
....................aaggauguuugacaaguaguu.......................................................................   3713    0   Mm2
....................aaggauguuugacGaguaguu.......................................................................   1       1   Mm2
....................aaggauguuuCacaaguaguu.......................................................................   3       1   Mm2
....................aaggauguuugacaaguaguG.......................................................................   4       1   Mm2
....................aaggauguuugacaagCaguu.......................................................................   16      1   Mm2
....................Uaggauguuugacaaguaguu.......................................................................   11      1   Mm2
....................aaggaCguuugacaaguaguu.......................................................................   1       1   Mm2
....................aaggauguuugacaaguGguu.......................................................................   7       1   Mm2
....................aaggGuguuugacaaguaguu.......................................................................   1       1   Mm2
....................Gaggauguuugacaaguaguu.......................................................................   9       1   Mm2
....................aaggauguuugacaUguaguu.......................................................................   1       1   Mm2
....................Caggauguuugacaaguaguu.......................................................................   2       1   Mm2
....................aaggauguuugacaagGaguu.......................................................................   9       1   Mm2
....................aaggauguuugacaaguaguA.......................................................................   6       1   Mm2
....................aaggauguuugacaGguaguu.......................................................................   1       1   Mm2
....................aaggauguuugacaaguaguC.......................................................................   1       1   Mm2
....................aaggCuguuugacaaguaguu.......................................................................   1       1   Mm2
....................aaggauguCugacaaguaguu.......................................................................   1       1   Mm2
....................aCggauguuugacaaguaguu.......................................................................   1       1   Mm2
....................aNggauguuugacaaguaguu.......................................................................   1       1   Mm2
....................aaUgauguuugacaaguaguu.......................................................................   2       1   Mm2
....................aaggauguuugacaaguUguu.......................................................................   9       1   Mm2
....................aaggauguuugacaaguCguu.......................................................................   8       1   Mm2
....................aaggauguuugacaagAaguu.......................................................................   8       1   Mm2
....................Naggauguuugacaaguaguu.......................................................................   1       1   Mm2
....................aaggauguuuAacaaguaguu.......................................................................   7       1   Mm2
....................aaggauguuugGcaaguaguu.......................................................................   2       1   Mm2
....................aaggauguuugGcaaguaguuu......................................................................   5       1   Mm2
....................aaggauguuugacaagGaguuu......................................................................   95      1   Mm2
....................aaggauguuuAacaaguaguuu......................................................................   60      1   Mm2
....................aaggaCguuugacaaguaguuu......................................................................   2       1   Mm2
....................aaggauguuugacaaguCguuu......................................................................   110     1   Mm2
....................aaggauguuugacUaguaguuu......................................................................   1       1   Mm2
....................aaggauguuugaUaaguaguuu......................................................................   4       1   Mm2
....................aaggauguuugacaaguaguuu......................................................................   32256   0   Mm2
....................aNggauguuugacaaguaguuu......................................................................   1       1   Mm2
....................aaggauguuAgacaaguaguuu......................................................................   4       1   Mm2
....................aaggauguuugacaagAaguuu......................................................................   70      1   Mm2
....................Gaggauguuugacaaguaguuu......................................................................   69      1   Mm2
....................Naggauguuugacaaguaguuu......................................................................   15      1   Mm2
....................aaggauguuugacaaguaguCu......................................................................   4       1   Mm2
....................aaggaAguuugacaaguaguuu......................................................................   1       1   Mm2
....................aaggauguuugacaagCaguuu......................................................................   120     1   Mm2
....................aaggauguuugacGaguaguuu......................................................................   6       1   Mm2
....................aaggauguuCgacaaguaguuu......................................................................   4       1   Mm2
....................aaggauguuugacaaguUguuu......................................................................   74      1   Mm2
....................aagCauguuugacaaguaguuu......................................................................   2       1   Mm2
....................aGggauguuugacaaguaguuu......................................................................   182     1   Mm2
....................aaggaNguuugacaaguaguuu......................................................................   4       1   Mm2
....................aUggauguuugacaaguaguuu......................................................................   4       1   Mm2
....................aaggauguuugacaaguaguGu......................................................................   4       1   Mm2
....................aaggauUuuugacaaguaguuu......................................................................   1       1   Mm2
....................aaggaugAuugacaaguaguuu......................................................................   5       1   Mm2
....................Uaggauguuugacaaguaguuu......................................................................   51      1   Mm2
....................aaAgauguuugacaaguaguuu......................................................................   3       1   Mm2
....................aaggauguuugacaaAuaguuu......................................................................   2       1   Mm2
....................aaggauguuugacaaguagAuu......................................................................   4       1   Mm2
....................aaggauguuugacaGguaguuu......................................................................   4       1   Mm2
....................aaggauguGugacaaguaguuu......................................................................   2       1   Mm2
....................aaUgauguuugacaaguaguuu......................................................................   5       1   Mm2
....................aaggauguCugacaaguaguuu......................................................................   1       1   Mm2
....................aaggauguuugacaUguaguuu......................................................................   8       1   Mm2
....................aaggauguuuCacaaguaguuu......................................................................   30      1   Mm2
```

```
                    Mature                              Star
aaccuugguauaugaaacguaaggauguuugacaaguaguuuugugcgucgacaaacugcuuuuugaacauacuccucguucuuuaccgagucugcuuauugcuucacgug
....................aaggauNuuugacaaguaguuu.....................................................................    2     1    Mm2
....................aaggauguuugCcaaguaguuu.....................................................................    1     1    Mm2
....................aaggaugGuugacaaguaguuu.....................................................................    2     1    Mm2
....................aaggGuguuugacaaguaguuu.....................................................................    5     1    Mm2
....................aaggauguAugacaaguaguuu.....................................................................    2     1    Mm2
....................aaggauCuuugacaaguaguuu.....................................................................    1     1    Mm2
....................aaggUuguuugacaaguaguuu.....................................................................    5     1    Mm2
....................aaggauguuugUcaaguaguuu.....................................................................    15    1    Mm2
....................aaggCuguuugacaaguaguuu.....................................................................    3     1    Mm2
....................aaggauguuugacaaguagGuu.....................................................................    3     1    Mm2
....................aaggauguuugacaaguaguuC.....................................................................    2     1    Mm2
....................aaggauguuugacaaguaUuuu.....................................................................    2     1    Mm2
....................aaggauguuugacaaguagCuu.....................................................................    7     1    Mm2
....................aaggauAuuugacaaguaguuu.....................................................................    3     1    Mm2
....................aaggaugCuugacaaguaguuu.....................................................................    2     1    Mm2
....................aaggauguuugacaaUuaguuu.....................................................................    4     1    Mm2
....................Caggauguuugacaaguaguuu.....................................................................    3     1    Mm2
....................aaggauguuugacaaguaguuG.....................................................................    25    1    Mm2
....................aagAauguuugacaaguaguuu.....................................................................    3     1    Mm2
....................aaggauguuugacaaguaguuA.....................................................................    39    1    Mm2
....................aaggauguuGgacaaguaguuu.....................................................................    1     1    Mm2
....................aaggauguuugacaaguGguuu.....................................................................    91    1    Mm2
....................aagUauguuugacaaguaguuu.....................................................................    2     1    Mm2
....................aaggauguuuUacaaguaguuu.....................................................................    28    1    Mm2
....................aaggauguuugacCaguaguuu.....................................................................    1     1    Mm2
....................aCggauguuugacaaguaguuu.....................................................................    3     1    Mm2
....................aaggauguuugacaaguaguAu.....................................................................    1     1    Mm2
....................aGggauguuugacaaguaguuuu....................................................................    5     1    Mm2
....................aaggauguuugacaUguaguuuu....................................................................    1     1    Mm2
....................aaggauguuugacaaguGguuuu....................................................................    11    1    Mm2
....................aaggCuguuugacaaguaguuuu....................................................................    1     1    Mm2
....................aaggauguuugacaagCaguuuu....................................................................    4     1    Mm2
....................aaggaCguuugacaaguaguuuu....................................................................    1     1    Mm2
....................aaggauguuugacaaguaUuuuu....................................................................    1     1    Mm2
....................aaggauguuugacaaguCguuuu....................................................................    6     1    Mm2
....................aaggauguuugacaaguaguuAu....................................................................    1     1    Mm2
....................aaggauguuugacaaCuaguuuu....................................................................    1     1    Mm2
....................aaggauguuugacaaguaguuuC....................................................................    1     1    Mm2
....................aaggauguuugacaaguaguuuA....................................................................    40    1    Mm2
....................aaggauguuugacaagGaguuuu....................................................................    3     1    Mm2
....................aaggauguuuCacaaguaguuuu....................................................................    3     1    Mm2
....................Uaggauguuugacaaguaguuuu....................................................................    4     1    Mm2
....................Naggauguuugacaaguaguuuu....................................................................    1     1    Mm2
....................aaggauguuuAacaaguaguuuu....................................................................    4     1    Mm2
....................aaggauguuugacaaguUguuuu....................................................................    6     1    Mm2
....................aCggauguuugacaaguaguuuu....................................................................    2     1    Mm2
....................aaggauguuugacaagAaguuuu....................................................................    3     1    Mm2
....................aaggauguuugacaaguaguuuG....................................................................    1     1    Mm2
....................aaggauguuugaAaaguaguuuu....................................................................    1     1    Mm2
....................Gaggauguuugacaaguaguuuu....................................................................    3     1    Mm2
....................aaggauguuugaUaaguaguuuu....................................................................    1     1    Mm2
....................aaggauguuugacaaguaguuuu....................................................................    1678  0    Mm2
....................aaggauguuuUacaaguaguuuu....................................................................    2     1    Mm2
....................aaggauguuugacaaguaguuuuU...................................................................    62    1    Mm2
....................aaggauguuugacaaguaguuuuUu..................................................................    3     1    Mm2
....................aaggauguuugacaaguaguuuAgu..................................................................    1     1    Mm2
.....................aggauguuugacaaguaguu......................................................................    1     0    Mm2
.....................aggauguuugacaaguagGuu.....................................................................    1     1    Mm2
.....................aggauguuugGcaaguaguuu.....................................................................    1     1    Mm2
.....................aggauguuugacaaguaguuu.....................................................................    20    0    Mm2
.......................gauguuugacaaguaguuu.....................................................................    1     0    Mm2
.......................................................acugcuuuuugaacauacuccu..................................    2     0    Mm2
.......................................................acugcuuuuugaacauacuccuc.................................    23    0    Mm2
.......................................................acugcuuuuugaacauaAuccuc.................................    1     1    Mm2
.......................................................acugcuuuuugaacauacuccucU................................    1     1    Mm2
........................................................cugcuuuuugaacauacuccuc.................................    3     0    Mm2

....................aaggauguuugacaagua.........................................................................    11    0    Mm1
....................aaggauguuugacaaguag........................................................................    12    0    Mm1
....................aaggauguuugacaaguagu.......................................................................    331   0    Mm1
```

```
                    Mature                             Star
aaccuugguauaugaaacguaaggauguuugacaaguaguuuugugcgucgacaaacugcuuuuugaacauacuccucguucuuuaccgagucugcuuauugcuucacgug
....................Uaggauguuugacaaguagu........................................................................   2      1     Mm1
....................aaggauguuugacaagCagu........................................................................   1      1     Mm1
....................aaggauguuugacaaguUgu........................................................................   1      1     Mm1
....................aaggauguuugacaaguCgu........................................................................   2      1     Mm1
....................aaggauguuugacaaguGgu........................................................................   1      1     Mm1
....................aGggauguuugacaaguagu........................................................................   6      1     Mm1
....................aaggauguuugacaagAagu........................................................................   1      1     Mm1
....................aaggauguuugacaagGagu........................................................................   1      1     Mm1
....................Uaggauguuugacaaguaguu......................................................................   5      1     Mm1
....................aaggauguuugacaaguaguG......................................................................   1      1     Mm1
....................Gaggauguuugacaaguaguu......................................................................   8      1     Mm1
....................aaggauguuugacaagAaguu......................................................................   5      1     Mm1
....................aaggauguuugacaagGaguu......................................................................   3      1     Mm1
....................aaggauguuuAacaaguaguu......................................................................   3      1     Mm1
....................aGggauguuugacaaguaguu......................................................................   7      1     Mm1
....................aaggauguuugacaaguGguu......................................................................   5      1     Mm1
....................aaggauguuugacUaguaguu......................................................................   2      1     Mm1
....................aaggauguuugacaaguaguu......................................................................   1391   0     Mm1
....................aaggauguuugacaagCaguu......................................................................   3      1     Mm1
....................Naggauguuugacaaguaguu......................................................................   1      1     Mm1
....................aaCgauguuugacaaguaguu......................................................................   1      1     Mm1
....................aaggauguuugacaaguUguu......................................................................   2      1     Mm1
....................aaggauguuuCacaaguaguu......................................................................   1      1     Mm1
....................aaggauguuugCcaaguaguu......................................................................   1      1     Mm1
....................aaggauguuugacaGguaguu......................................................................   1      1     Mm1
....................aaggauguuugacaaguCguu......................................................................   6      1     Mm1
....................aaggauguuugUcaaguaguu......................................................................   1      1     Mm1
....................aaggauUuuugacaaguaguu......................................................................   1      1     Mm1
....................aaggauguuugacaUguaguu......................................................................   1      1     Mm1
....................aaggauguuuUacaaguaguu......................................................................   2      1     Mm1
....................aaggauAuuugacaaguaguu......................................................................   1      1     Mm1
....................aaggauguuugacaagGaguuu.....................................................................   8      1     Mm1
....................aaggauguuugaUaaguaguuu.....................................................................   2      1     Mm1
....................aaggauguuugacaaguCguuu.....................................................................   19     1     Mm1
....................aaggaAguuugacaaguaguuu.....................................................................   1      1     Mm1
....................Gaggauguuugacaaguaguuu.....................................................................   7      1     Mm1
....................aaggauguuugacaaguaUuuu.....................................................................   1      1     Mm1
....................aaggauguuugacaagAaguuu.....................................................................   8      1     Mm1
....................aaggaugAuugacaaguaguuu.....................................................................   1      1     Mm1
....................aagUauguuugacaaguaguuu.....................................................................   1      1     Mm1
....................aaggauguuugacaUguaguuu.....................................................................   2      1     Mm1
....................aaggauguuCgacaaguaguuu.....................................................................   2      1     Mm1
....................aaggauguCugacaaguaguuu.....................................................................   2      1     Mm1
....................Uaggauguuugacaaguaguuu.....................................................................   8      1     Mm1
....................aaggauguuugGcaaguaguuu.....................................................................   1      1     Mm1
....................aaggauguuugacaaguGguuu.....................................................................   24     1     Mm1
....................aaCgauguuugacaaguaguuu.....................................................................   1      1     Mm1
....................aaggauguuugacUaguaguuu.....................................................................   1      1     Mm1
....................aaggauguuuUacaaguaguuu.....................................................................   4      1     Mm1
....................aaggauguuugacaGguaguuu.....................................................................   2      1     Mm1
....................aaNgauguuugacaaguaguuu.....................................................................   1      1     Mm1
....................aGggauguuugacaaguaguuu.....................................................................   5      1     Mm1
....................aUggauguuugacaaguaguuu.....................................................................   1      1     Mm1
....................aaggauguuugacaaguagCuu.....................................................................   1      1     Mm1
....................aaggauguuugacGaguaguuu.....................................................................   4      1     Mm1
....................aaggauguuugacaaguUguuu.....................................................................   17     1     Mm1
....................aaggauguAugacaaguaguuu.....................................................................   1      1     Mm1
....................aaggCuguuugacaaguaguuu.....................................................................   1      1     Mm1
....................Naggauguuugacaaguaguuu.....................................................................   1      1     Mm1
....................aaggauguuugacaaguaguuu.....................................................................   6325   0     Mm1
....................aaggauguuugacaagCaguuu.....................................................................   15     1     Mm1
....................aaggauguuuAacaaguaguuu.....................................................................   8      1     Mm1
....................aaggaugCuugacaaguaguuu.....................................................................   1      1     Mm1
....................aaAgauguuugacaaguaguuu.....................................................................   3      1     Mm1
....................aaggauguuugacaaguaguuG.....................................................................   2      1     Mm1
....................aaggauguuugacaaguaguCu.....................................................................   4      1     Mm1
....................aaggauguuuCacaaguaguuu.....................................................................   10     1     Mm1
....................aagCauguuugacaaguaguuu.....................................................................   1      1     Mm1
....................aaggauguuugacaaguaguuA.....................................................................   32     1     Mm1
....................aaggaNguuugacaaguaguuu.....................................................................   1      1     Mm1
```

```
                    Mature                             Star
aaccuugguauaugaaacguaaggauguuugacaaguaguuuugugcgucgacaaacugcuuuuugaacauacuccucguuucuuuaccgagucugcuuauugcuucacgug
....................aagAauguuugacaaguaguuu......................................................................    2     1     Mm1
....................aaggUuguuugacaaguaguuu......................................................................    1     1     Mm1
....................aaggauguuugaAaaguaguuu......................................................................    1     1     Mm1
....................aaggauguuugacaagGaguuuu.....................................................................    1     1     Mm1
....................aaggauguuuAacaaguaguuuu.....................................................................    1     1     Mm1
....................aaggaAguuugacaaguaguuuu.....................................................................    1     1     Mm1
....................aaggauguuugacaaguaguuCu.....................................................................    1     1     Mm1
....................aaggauguuugacaaguagAuuu.....................................................................    1     1     Mm1
....................aaggauguuuCacaaguaguuuu.....................................................................    1     1     Mm1
....................aaggauguuugacaaguCguuuu.....................................................................    1     1     Mm1
....................aaggauguuugacaagAaguuuu.....................................................................    2     1     Mm1
....................aaggauguuugacaaguaguuuu.....................................................................    362   0     Mm1
....................aaggauguuugacaagCaguuuu.....................................................................    2     1     Mm1
....................aaggauguuugacaaguaguuuA.....................................................................    6     1     Mm1
....................aUggauguuugacaaguaguuuu.....................................................................    1     1     Mm1
....................aaggCuguuugacaaguaguuuu.....................................................................    1     1     Mm1
....................Caggauguuugacaaguaguuuu.....................................................................    1     1     Mm1
....................aaggauguuugacaGguaguuuu.....................................................................    2     1     Mm1
....................aaggauguuugacaaguaguuuuU....................................................................    16    1     Mm1
.....................aggauguuugacaaguaguuu......................................................................    4     0     Mm1
```
